# Regulation of autophagy by polyphenolic compounds as a potential therapeutic strategy for cancer

**DOI:** 10.1038/cddis.2014.467

**Published:** 2014-11-06

**Authors:** N Hasima, B Ozpolat

**Affiliations:** 1 Department of Experimental Therapeutics, The University of Texas MD Anderson Cancer Center, 1515 Holcombe Boulevard, Unit 422, Houston, TX 77030, USA; 2 Institute Science Biology, Faculty of Science, University of Malaya, Kuala Lumpur 50603, Malaysia; 3 Center for Research in Biotechnology for Agriculture, University of Malaya, Kuala Lumpur 50603, Malaysia; 4 Center for RNA Interference and Non-Coding RNAs - Red and Charline McCombs Institute for the Early Detection and Treatment of Cancer, The University of Texas MD Anderson Cancer Center, 1515 Holcombe Boulevard, Unit 422, Houston, TX, USA

## Abstract

Autophagy, a lysosomal degradation pathway for cellular constituents and organelles, is an adaptive and essential process required for cellular homeostasis. Although autophagy functions as a survival mechanism in response to cellular stressors such as nutrient or growth factor deprivation, it can also lead to a non-apoptotic form of programmed cell death (PCD) called autophagy-induced cell death or autophagy-associated cell death (type II PCD). Current evidence suggests that cell death through autophagy can be induced as an alternative to apoptosis (type I PCD), with therapeutic purpose in cancer cells that are resistant to apoptosis. Thus, modulating autophagy is of great interest in cancer research and therapy. Natural polyphenolic compounds that are present in our diet, such as rottlerin, genistein, quercetin, curcumin, and resveratrol, can trigger type II PCD via various mechanisms through the canonical (Beclin-1 dependent) and non-canonical (Beclin-1 independent) routes of autophagy. The capacity of these compounds to provide a means of cancer cell death that enhances the effects of standard therapies should be taken into consideration for designing novel therapeutic strategies. This review focuses on the autophagy- and cell death-inducing effects of these polyphenolic compounds in cancer.

## Facts

- Natural polyphenolic compounds that are present in our diet, such as rottlerin, genistein, quercetin, curcumin, and resveratrol can alter the effects of signaling pathways and induce cell death not only via apoptosis but also via autophagy. Thus, these compounds could be used as a co-therapy with standard therapies in cancer.
- These compounds can trigger type II PCD via various mechanisms through the canonical (Beclin-1 dependent) and non-canonical (Beclin-1 independent) routes of autophagy.
- Rottlerin or its related analogs may be used in the development of novel agents for the induction of autophagic cell death as it has been proven, pharmacodynamically in a mice xenograft model, to be efficiently absorbed in cells and tissues against pancreatic cancer.
- Genistein induced autophagy due to changes in apoptotic signaling, which is beneficial against chemoresistance usually seen in cancer cells.
- Quercetin induced extensive autophagy and subsequent death in cancer cells mediated by the inhibition of proteasomal activity and mTOR signaling.
- Curcumin induced G_2_/M arrest and autophagy in malignant glioma cells through the inhibition of the Akt/mTOR/p70S6K and activation of the extracellular signal-regulated kinase (ERK)1/2 pathways, which implied that cell death via autophagy, might be pathway specific.
- Resveratrol induced cell death through autophagy in five ovarian cancer cell lines, suggesting that it may be effective treatment in apoptosis-resistant ovarian cancer. Autophagy can be induced with acute exposure to resveratrol, whereas prolonged exposure activates a caspase-mediated cell death pathway.

## Open Questions

- The induction of cellular senescence was accompanied by autophagy in colon cancer cells with an increase in Beclin-1 and p62/SQSTM1 protein levels. Therefore, the functional link between senescence and autophagy in these curcumin-treated cancer cells should be further investigated.
- Autophagy inhibitors may have the potential to enhance resveratrol antitumor efficacy.
- The ability of natural polyphenolic compounds to induce autophagic cell death that enhances the effects of standard therapies should be taken into consideration for designing novel therapeutic strategies.
- Combining FDA (food and drug activation)-approved drugs with these polyphenolic compounds such as rottlerin, genistein, quercetin, curcumin, and resveratrol may provide novel therapeutic strategies in the treatment of cancer.

**Figure 1:**
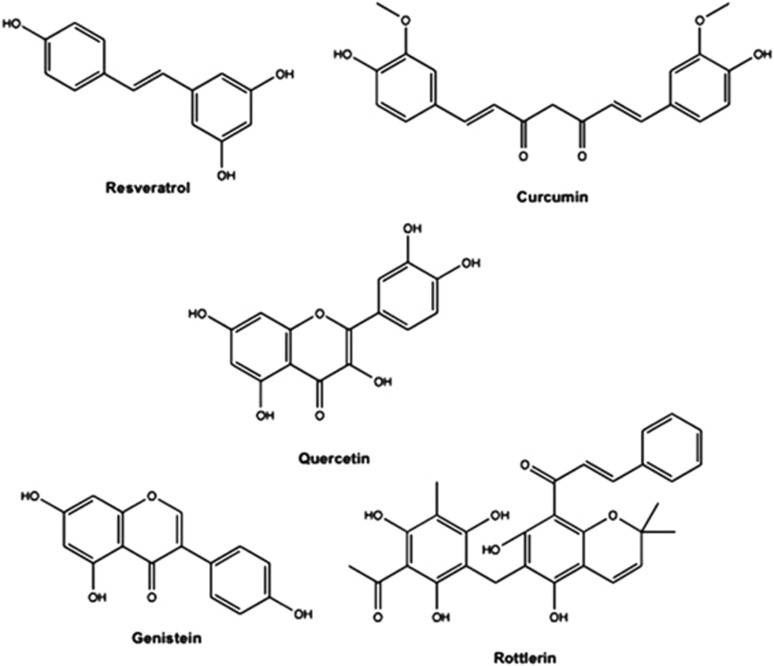
Chemical structures of autophagy-inducing polyphenolic compounds

**Figure 2:**
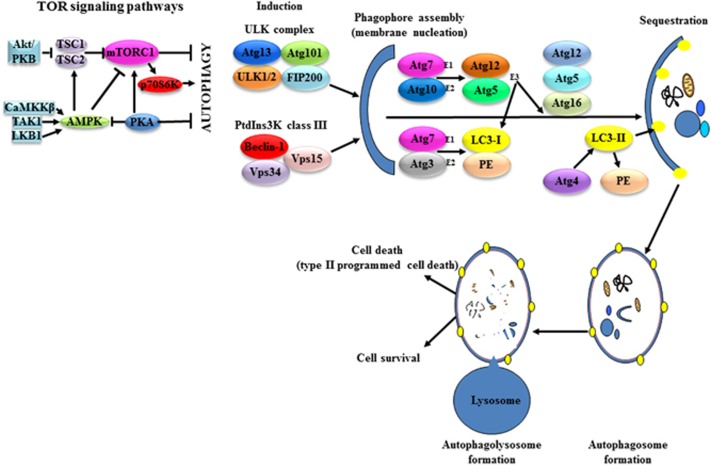
The major signaling pathway regulation and core machinery of autophagy. Various pathways regulate autophagy either positively or negatively. Most of these pathways, including AMPK and PKA, merge at mTORC1. PKA directly activates mTORC1, inactivating both AMPK and autophagy. AMPK negatively regulates mTORC1 in two ways, either directly or by activating the TSC2 protein. The mTORC1 substrate p70S6K is a positive regulator of autophagy. Another important upstream factor is Akt/PKB, a negative regulator of the TSC1/2 complex. The core machinery includes the processes of induction, phagophore assembly (membrane nucleation), sequestration, autophagosome formation, and autophagolysosome formation. The process of induction involves initiation of the UNC-51-like kinase complex members ULK 1/2, Atg13, Atg101, and FIP200. Activation of the PtdIns3K complex (Beclin-1, Vps34, and Vps15) is an essential step in phagophore assembly (membrane nucleation). The E1-like enzyme Atg7 activates both Atg12 and LC3-I, and the E2-like enzyme activates Atg10 (for Atg12) and Atg3 (for LC3-I). Atg12 is conjugated to Atg5 and the Atg12-Atg5 complex acts as an E3 ubiquitin ligase to catalyze the conjugation of LC3-I to the lipid phosphatidylethanolamine (PE) in the process of sequestration. The subsequent autophagosome formation is dependent on the Atg12-Atg5-Atg16 complex. After autophagosome completion, the Atg12-Atg5-Atg16 complex dissociates from autophagosomes to allow Atg4 access to LC3-II-PE for deconjugation. When the autophagosome is completed, it fuses with lysosome to form an autophagolysosome in which the cytosolic macromolecules, proteins, and organelles will either be degraded by acid hydrolases in what is known as type II programmed cell death or involved in a survival mechanism to be released back into the cytosol with the help of permeases against cellular stress. Akt/PKB, protein kinase B; AMPK, adenosine monophosphate-activated protein kinase; CaMKKβ, calmodulin kinase kinase β Atg, autophagy-related genes; FIP200, focal adhesion kinase (FAK) family-interacting protein of 200kD; LC3, microtubule-associated protein 1 light chain 3; LKB1, liver kinase B1; mTORC1, mammalian target of rapamycin complex 1; PE, phosphatidylethanolamine; p70S6K, p70S6 kinase; PKA, protein kinase A; PtdIns3K, phosphatidylinositol 3-kinase; TAK1, TGFβ-activated kinase 1; TSC1/2, tuberous sclerosis complex 1/2; ULK 1/2, UNC-51–like kinases 1/2; Vps 15/34, vacuolar protein sorting 15/34

**Figure 3:**
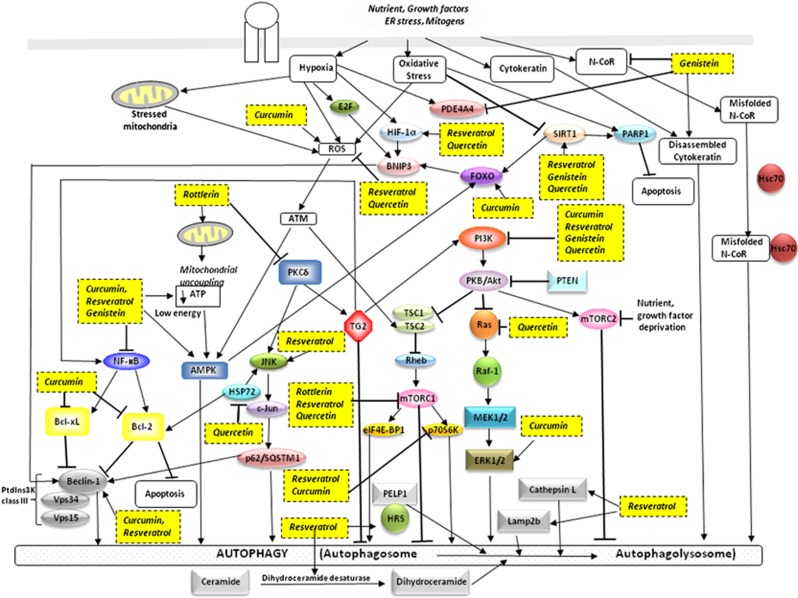
Polyphenols promote autophagic cell death in cancer cells. The polyphenolic compounds rottlerin, genistein, quercetin, curcumin, and resveratrol induce cell death via autophagy through inhibition or activation of multiple signaling pathways and cellular targets in cancer cells. The common pathways involved are PI3K/Akt/mTOR, NF-κB, MEK/ERK, PKCδ/TG2, JNK/p62/SQSTM1, and AMPK/TSC2. Akt/PKB, protein kinase B; AMPK, adenosine monophosphate-activated protein kinase; ATM, ataxia telangiectasia mutated; ATP, adenosine triphosphate; Bcl-2, B-cell lymphoma 2; Bcl-XL, B cell lymphoma-extra large; BNIP3, Bcl-2 and nineteen KD interacting protein-3; eIF4E-BP1, eukaryotic translation initiation factor 4E-binding protein 1; ERK1/2, extracellular signal-regulated kinase 1/2; FOXO, forkhead transcription factors; HIF-1*α*, Hypoxia-inducible factor 1-alpha; HRS, hepatocyte growth factor-regulated tyrosine kinase substrate; Hsc70, 70-kDa heat shock protein family expressed constitutively; HSP72, 70-kDa heat shock protein family stress- and heat shock-induced; JNK, c-Jun N-terminal kinase; Lamp2b, lysosome-associated membrane proteins 2b; MEK1/2, mitogen-activated protein kinase 1/2; mTORC1/2, mammalian target of rapamycin complex 1/2; N-CoR, putative corepressor; NF-*κ*B, nuclear factor-kappa B; PARP1, poly(ADP-ribose) polymerase 1; PDE4A4, cyclic AMP phosphodiesterase-4A4; PELP1; proline-, glutamic acid-, and leucine-rich protein-1; PI3K, phosphatidylinositol 3-kinase; PKCδ, protein kinase C-delta; PTEN, phosphatase and tensin homolog deleted on chromosome ten; p62/SQSTM1, p62 protein /sequestosome 1; p70S6K, p70 ribosomal protein S6 kinase; Raf-1, oncoprotein activated by Ras; Ras, oncoprotein; Rheb, Ras homolog enriched in brain; ROS, reactive oxygen species; SIRT1, sirtuin 1; TG2, transglutaminase 2; TSC1/2, tuberous sclerosis complex 1/2; Vps 15/34, vacuolar protein sorting 15/34

Natural plant-derived polyphenols are chemical substances characterized by the presence of more than one phenol unit per molecule. They are present in some foods and have been shown to exert anticancer properties. Some important examples are rottlerin, genistein, quercetin, curcumin, and resveratrol, all of which have been shown to induce autophagy death in various cancer cells (Figures 1,2,3 and Table 1).

Macroautophagy (hereafter called autophagy) is a lysosomal catabolic process conserved through evolution in eukaryotes for degrading long-lived proteins, macromolecules, and organelles from the cytoplasm.^1, 2^ Autophagy has different roles in normal and cancer cells, especially in the tumor microenvironment. Although genetic evidence indicates that autophagy functions as a tumor suppressor in normal cells, it can promote survival of established tumors in the presence of cellular stress factors, including nutrient deprivation, hypoxia, metabolic, and therapy-induced stress.^3^ If excessively induced, autophagy can lead to a non-apoptotic form of programmed cell death (type II PCD) which is caspase independent. Autophagy may be seen in cells that have a high threshold for induction of apoptosis or defective apoptotic machinery, such as inappropriate regulation of pro- and antiapoptotic Bcl-2 family member proteins.^4^

Autophagy requires the sequestration of cytoplasmic content or organelles through the formation of double-membrane vesicles, controlled by autophagy-related genes (*ATG*) and their protein products. Beclin-1, one of the major Atg proteins (its counterpart in yeast is known as Atg6), controls vital steps in the autophagic pathway. It is interesting that autophagy can be regulated by various canonical and non-canonical pathways, some involving Beclin-1,^5, 6^ others Beclin-1 dependent and Atg5/Atg7 independent,^7^ Beclin-1/Vps34 independent,^8, 9^ or Beclin-1 independent and Vps34 dependent.^10, 11^ Studies characterizing metabolic cascades known to have a role in autophagy involved common pathways, such as PI3K/Akt/mTOR, NF-*κ*B, MAPK/MEK/ERK, protein kinase C delta (PKCδ)/ transglutaminase 2 (TG2), JNK/p62 /SQSTM1, and AMPK/TSC2, are reviewed here to gain insight into the regulation of autophagy (Table 1 and Figure 3).

In this review, which is an update to previous reports by our group,^3, 12, 13^ we look into the effects of five common dietary polyphenols – rottlerin, genistein, quercetin, curcumin, and resveratrol – on autophagy in several cancer types and their regulation of the various metabolic cascades that leads to the induction or inhibition of this alternative cell death mechanism.

## Autophagic pathways

Autophagy is a multistep process involving induction, phagophore formation, sequestration, formation of autophagosome, and finally its fusion with lysosome to form autophagolysosome, which then induces either the death or survival pathway (Figure 2). The autophagic pathway dependent on Beclin-1 defines the canonical pathway; the other, independent of Beclin-1, is accepted as non-canonical autophagy. The presence of non-canonical autophagy means that autophagy can be triggered in cells in which the expression of Beclin-1 is too low to induce canonical autophagy.

### Canonical/classic pathway

*Beclin-1*, an autophagy-promoting gene, was determined to function as a tumor suppressor after it was shown to be deleted in breast, ovarian, and prostate cancers.^5^ Beclin-1 forms a core complex known as PtdIns3K class III with Vps34 and Vps15 regulatory subunits (Figures 2 and 3), and this complex has been shown to promote autophagy by mediating autophagosome nucleation.^14^ Within the Beclin-1-dependent autophagy are two alternative downstream pathways, the more common, Atg5/Atg7 dependent and the other, Atg5/Atg7 independent.^7^

### Non-canonical/non-classic pathway

In contrast to canonical, non-canonical autophagy is a process that does not require the entire set of Atg proteins to form the autophagosome. This alternative form of autophagy, which is independent of Beclin-1 and, typically Vps34, has been described elsewhere.^8, 15^ It is important to note that Beclin-1-independent autophagy does not always imply independence from Vps34.^10, 11^ A common hallmark of this Beclin-1 independent autophagy is its dependence on the activity of the UNC-51 like kinase 1/2 complex to induce autophagy and LC3 for phagophore formation (Figures 2 and 3). The stimuli that trigger these forms of autophagy and the various interacting proteins involved in the formation and maturation of autophagosomes are not completely understood.

## Autophagy and cancer

Defects in autophagy alter cells' metabolic state and their capacity for protein degradation, and are associated with various disease conditions.^16, 17, 18^ Malignant cells often display defective autophagic activities compared with their normal counterparts. However, the role of autophagy in cancer is paradoxical in normal and malignant cells, and available data suggest that autophagy can promote tumor growth by helping tumor cells survive and inhibit tumor growth through its tumor-suppressor function in normal cells.^19^ Removal of damaged proteins and organelles may prevent cancer initiation while allowing established tumors to adapt to nutrient-deprived or hypoxic conditions during cancer progression.^20^

Initial genetic evidence in mice suggested that *Beclin-1* functions as a haplo-insufficient tumor suppressor and that its mono-allelic deletion leads to spontaneous tumors and upon re-expression, it restores autophagy and suppresses tumorigenesis.^21, 22^ Although Beclin-1 and LC3 are important mediators of autophagy, other molecules and signaling pathways (for example, p53, PI3K/AKT/mTOR) require critical examination to determine their roles in cells' autophagic capacity toward cell death or survival following various triggers.

On the other hand, the antiapoptotic Bcl-2 family members from the endoplasmic reticulum and not the mitochondria, such as Bcl-2 and Bcl-xL, inhibit autophagy by binding to the BH3 domain of Beclin-1.^23^ Furthermore, recent data suggest that the oncogenic effect of Bcl-2 arises from its ability to inhibit autophagy but not apoptosis. Inhibition of Bcl-2 leads to autophagic cell death in MCF7 breast cancer cells.^24^ Therefore these evidences suggest that modulating autophagy may be important in designing anticancer therapies.^22^ Finally, studies suggest that the genetic makeup of cells will determine its fate in terms of undergoing autophagy and response to standard or novel therapies.

## Signaling pathways regulating autophagy

Disruption of the normal balance between pro- and anti-autophagic signaling pathways is linked to cancer and other diseases. Several signaling pathways kinases, such as mTOR, AMPK, PI3Ks, MAPKs (ERK and JNK), and PKC, respond to various external factors and are often dysregulated in cancer (Figure 3). These kinases may be part of an energy-sensing mechanism and stress response. For example, the mTOR kinase is an important repressor of autophagy and controller of cell growth and proliferation. Therefore, it is not surprising that it is regulated by multiple signaling pathways; it is activated by the Akt/PKB proto-oncogenic pathway and often upregulated in various cancer types.^25^

There are two mTOR complexes, mTORC1 and mTORC2, which are regulated differently even though they are both induced in response to nutrient starvation, stress, and reduced growth factor signaling. TSC 1/2, an inhibitor of mTOR, is the gateway by which other signaling pathways influence mTOR activity. Alexander *et al.*^26^ provided evidence that the ATM protein signals to TSC2 via the AMPK metabolic pathway in the cytoplasm to repress mTORC1 in response to reactive oxygen species (ROS), thereby inducing autophagy. Alternatively, the mTOR kinase can be activated by Akt independently of TSC 1/2 and can be regulated by MAPKs. mTORC2 is rapamycin insensitive and activates Akt by phosphorylation, to contribute to another route in the regulation of autophagy.^25, 27^

Wong *et al.*^28^ observed the activation of ERK and JNK as upstream effectors controlling autophagy induced by ROS production. Wang *et al.*^28^ reported a non-canonical MEK signaling pathway positioned downstream of AMPK and upstream of TSC that mediates autophagy via regulation of Beclin-1.^6^ Puissant and Auberger^29^ observed the activation of AMPK/mTOR- and JNK-mediated p62/SQSTM1 in triggering autophagic cell death. AMPK, which promotes autophagy by activating TSC 1/2 and suppressing mTOR, has been implicated in an energy check point because of its roles in phosphorylating p53 and inducing cell cycle arrest following energy deprivation.^30^ We previously reported that PKCδ positively regulates the expression of TG2,^13, 14^ which leads to the suppression of autophagy through various downstream pathways, including NF-*κ*B, PI3K/Akt/mTOR/p70S6K and Bcl-2 anti-autophagic/apoptotic protein.^3, 12^ Hence, studies suggest that activated or inhibited signaling pathways that are involved in the regulation of autophagy will direct it in cancer cells.

## Clinical significance of autophagy and its modulation for treatment of cancer

The observations in clinical trials showed that Beclin-1 expression is altered in some cancers, overexpressed in others (intrahepatic cholangiocarcinoma and gastric tumors), whereas underexpressed in several solid tumors (breast, ovarian, cervical, lung, brain, liver, esophageal, gastric, and pancreatic) and osteosarcomas.^22, 31, 32, 33, 34, 35, 36^ *Beclin-1* gene is found to be deleted in ovarian, breast, and prostate cancers, suggesting that reduced autophagic capacity is usual in some commonly diagnosed cancers.^37, 38^ Apparently, the expression of LC3 and Beclin-1 is associated with cancer stage, low expression levels being associated with poorly differentiated tumors and more advanced clinical stage of disease. Examples of this correlation of Beclin-1 level with clinical cancer stage include ovarian,^32^ esophageal squamous cell carcinoma,^39^ chondrosarcoma,^40^ and lymphomas,^41, 42^ whereas no such connection has been found in cervix,^43^ nasopharynx^44^ or adenoid cystic carcinoma.^44^ More importantly, many chemotherapeutic agents, such as cisplatin, plant alkaloids, antimetabolites,^45, 46^ tyrosine kinase inhibitors,^47, 48^ and radiotherapy^49^ induce autophagy. However, it remains to be determined whether the effectiveness of these therapies is dependent on hyper or under functional autophagy.^20^

Currently, >30 clinical trials are investigating the effects of autophagy in combination with cytotoxic chemotherapy or targeted agents in various human cancers.^50^ For example, chloroquine and hydroxychloroquine, routinely used for the treatment of diverse diseases, inhibit lysosomal acidification and prevent autophagy. In cancer treatment, chloroquine is often used in combination with chemotherapeutic drugs, such as cisplatin or PI3K inhibitor, LY294002 or the mTOR inhibitor, rapamycin. With all these agents, however, sensitization occurs independently of autophagy inhibition, and was not mimicked by Atg12, Beclin-1 knockdown, or bafilomycin treatment, and occurred even in the absence of Atg12.^51^ The results of these clinical trials are critical for better understanding the process and role of autophagy in tumor biology and to validate the strategy of targeting autophagy to enhance therapeutic benefits to patients.

## Cell death by autophagy

Apoptosis (type I PCD) and necrosis (type III PCD) are well-known mechanisms of cell death induced by anticancer therapies. Recent studies have shown a non-apoptotic form of programmed death called autophagy, which is termed type II PCD and is often caspase independent.^52^ In both apoptosis and autophagy, the degraded cells are disposed by phagocytosis without an inflammatory response, in contrast to the extensive cellular disintegration and subsequent inflammation that occur in necrosis.

Studies have shown that there is a complex interplay between the apoptotic and autophagic processes. Autophagy may precede apoptosis or be induced simultaneously depending on the genetic context and cellular background of the cells. Oligomerized caspase-8 binds autophagosome membrane, leading to its activation and creating a mechanism for transition from apoptosis to autophagy.^53^ Experimental findings suggest that activated caspase-3 can cleave Beclin-1, producing a fragment that translocates to mitochondria and induces apoptosis.^54^

Other studies indicate that the Bcl-2 family of proteins not only regulates apoptosis but also controls cell death that depends on the autophagy genes.^24^ Cytotoxic signals can induce autophagy in apoptosis resistant cells, such as, those expressing high levels of Bcl-2 or Bcl-xL, lacking Bax and Bak, or exposed to pan-caspase inhibitors, such as, zVAD-fmk, suggesting autophagy to act as a default mechanism leading to cell death.^4^ For instance, although embryonic fibroblasts from Bax/Bak double-knockout mice are resistant to apoptosis, they can undergo autophagic death after stimulation.^4^ This non-apoptotic cell death was suppressed by autophagy inhibitors, such as, 3-MA, bafilomycin, or hydroxyl-choloroquine and or genetic silencing of autophagic genes (for example, *ATG5, ATG7*, *or Beclin-1*).^4^ This indicates that the autophagic process has a significant role in caspase-independent cell death. Further studies supported these findings when apoptosis-resistant Bax−/− and Bak−/− knockout fibroblast cells underwent autophagic death following induction, such as, starvation, growth factor withdrawal, chemotherapy (etoposide), or radiation.^55^ In addition, the knockdown of ATG5 or Beclin-1 in cancer cells has shown marked reduction in cell death and autophagic effects in response to death stimuli, with no sign of apoptosis.^12^

Autophagy has important roles in both, maintenance of cellular homeostasis under regular growth conditions and protection of cell viability under stress. In the housekeeping pathway, autophagy removes sources of ROS such as damaged or aggregated proteins and organelles to prevent tumor initiation via suppression of oxidative and genotoxic stress.^56, 57^ Under stress, however, autophagy supports tumor cell survival by providing substrates for mitochondrial metabolism.^58^ *In situations* of defective mitophagy, autophagy-deficient cells would accumulate damaged mitochondria and deregulate ROS levels, which have been suggested to contribute to induction of tumors.^59^ Kaminskyy *et al.*^60^ showed that autophagy suppression led to the inhibition of proliferation of non-small cell lung carcinoma cells and sensitized them to cisplatin-induced caspase-dependent and -independent apoptosis by stimulation of ROS formation.

**Figure 4:**
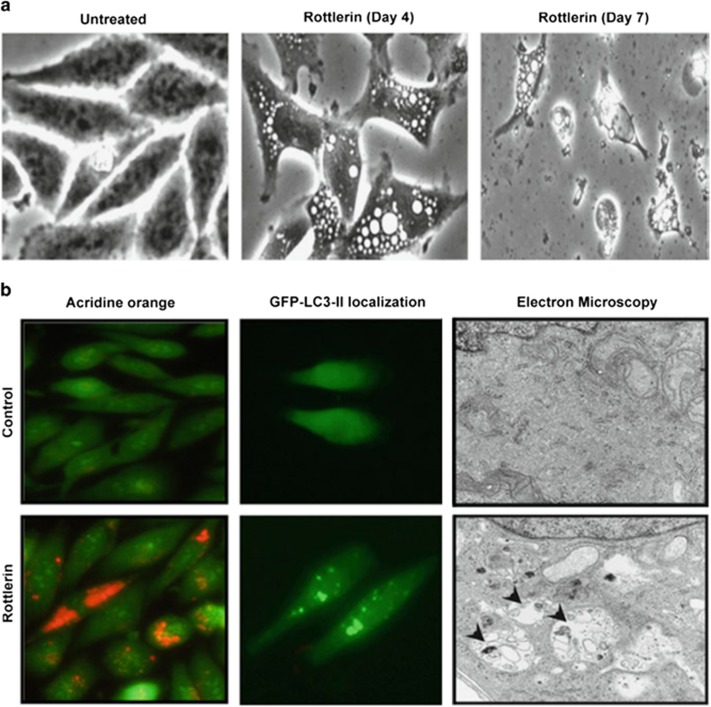
Rottlerin induced massive autophagy and cell death in pancreatic cancer cells. (**a**) Morphological changes and formation of autophagic vacuoles in MDA-Panc28 pancreatic cancer cells that were treated with 4 *μ*M rottlerin. Microphotographs were taken using a phase-contrast microscope ( × 300 magnification). Left panel, untreated cells at 48 h; center panel, cells treated for 4 days; and right panel, cells treated for 7 days. (**b**) MDA-Panc28 cells were treated with Rottlerin for 48 h and then were stained with acridine orange for detection of acidic vesicular organelles by fluorescent microscopy (left panels) and localization of LC3-II at autophagosomes in cells after transfection with GFP-LC3 plasmid (middle panels).^12, 13^ Green fluorescence indicates cytoplasm and nucleus, whereas red fluorescence shows acidic vesicular organelles. Right panels, electron micrographs showing the ultrastructure of rottlerin-treated cells (4 M). Numerous autophagic vacuoles, indicated by arrows, were observed in the rottlerin-treated cells

Autophagy leading to cell death is induced in various cancer cells in response to treatment by several polyphenolic compounds,^61^ including rottlerin (Figure 4),^12^ curcumin,^62^ resveratrol,^63^ genistein,^64, 65^ and quercetin,^66^ as well as some chemotherapeutic agents, such as cytosine arabinoside,^67^ etoposide,^4^ and staurosporine,^68^ and growth factor deprivation.^67^

## Polyphenolic compounds and autophagic cell death

Polyphenols are a structural class of natural organic chemicals characterized by the presence of large multiples of phenol structural units. These compounds found in foods possess anticancer activities in their ability to alter the effects of signaling pathways and induce cell death not only via apoptosis but also autophagy.^69, 70^

## Rottlerin

Rottlerin (5, 7-dihydroxy-2, 2-dimethyl-6-(2, 4, 6-trihydroxy-3-methyl-5-acetylbenzyl)-8-cinnamoyl-1, 2-chromine), also called mallotoxin, is isolated from *Mallotus phillippinensis* (the monkey-faced tree). Rottlerin induces autophagy via three distinct mechanisms, that is, through PKCδ/TG2, PKCδ-independent, and mTORC1 pathways, in pancreatic cancer, fibrosarcoma, and breast cancer, respectively (Table 1and Figure 3).

Rottlerin displayed antioxidant properties and inhibitory effect on NF-*κ*B in breast and colon cancer cells.^71^ In fact, in these cells, expressions of PKCδ and TG2 led to activation of NF-*κ*B,^72, 73^ whereas inhibition led to induction of autophagy death.^74^ Importantly, rottlerin is accepted widely as a PKCδ-selective inhibitor.

The first evidence of massive autophagy induction leading to death only in cells with PKCδ via induction of TG2 was reported in pancreatic cancer cells.^12, 13^ However, recent studies suggested that rottlerin can also induce apoptosis through PKCδ-independent mechanisms in fibrosarcoma cells.^75^ It was suggested that the early autophagy might serve as a survival mechanism against late apoptosis in this cancer type.^76^

Rottlerin was able to inhibit mTORC1 signaling, via its negative regulator, TSC2 to induce autophagosome accumulation in breast cancer cells in nutrient-rich conditions.^77^

Rottlerin inhibition of NF-κB was able to induce AMPK induction, which led to significantly reduced cellular ATP levels and induction of autophagy in cancer cells.^78^ AMPK can also activate the cyclin-dependent kinase inhibitor, p27, by a mechanism involving the SIRT1/FOXO pathway to induce autophagy.^79^

Overall, rottlerin-induced autophagy may involve multiple signaling pathways and cellular mechanisms for induction of autophagy and eventual cell death. However, the most important factor determining the fate of cells is probably the cellular context, increased apoptotic threshold/resistance and activated/inhibited signaling pathways. Thus rottlerin or its related analogs may be used in the development of novel agents for induction of autophagic cell death as it has been proven pharmacodynamically in a mice xenograft model to be efficiently absorbed in cells and tissues against pancreatic cancer.^80^

## Genistein

Genistein (4′, 5, 7-trihydroxyisoflavone), a naturally occurring isoflavonoid found in soy products, has been shown to have anticancer properties. It has the capacity to induce cell death through both apoptosis^81^ and autophagy.^82^ Thus, genistein may be beneficial against chemoresistance owing to changes in apoptotic signaling (Table 1).

It has been shown to completely protect the cytokeratin network in stress, nutrient- and growth factor-deprived environments. Various studies showed its ability to overcome the disruptive effects of okadaic acid, a strong inhibitor of autophagy, on the organization of the cytoskeleton and cytokeratin in rat hepatocytes.^64, 65^ This is of consequence, as the cytokeratin filaments are involved in the development of autophagy.

Genistein has been shown to be cytotoxic in ovarian cancer cells with mechanism of death involving not only apoptosis but also autophagy.^82^ Treatment markedly inhibited glucose uptake in these cells, and methyl pyruvate, the substrate for oxidative phosphorylation and fatty acid synthesis, could rescue cells from genistein-induced autophagy. Gossner *et al.*^82^ also showed that treatment reduced levels of phosphorylated Akt. This may have contributed to limiting glucose utilization, which suggested that a starvation-like signaling response would eventually lead to autophagy death. Christian *et al.*^83^ reported its ability to inhibit both PKC and ERK inhibitors via inhibition of PDE4A4 aggregate formation to activate autophagy in ovarian cancer cells. These authors suggested that, as PDE4A4 aggregates are neither autophagosomes nor aggresomes and constitutively co-immunoprecipitated with the p62 protein (SQSTM1). Therefore, inhibiting their formation would be beneficial in ensuring induction of autophagy, as p62 interacts with LC3, which is critical for membrane encapsulation in autophagosomes.^84^

Ali *et al.*^85^ reported evidence for genistein inhibition of N-CoR misfolding, an important component in the activation of the oncogenic survival pathway in non-small cell lung carcinoma, which was found to be associated with Hsc70, a molecular chaperone in autophagy (Figure 3).

Although, genistein can induce autophagy and apoptotic death in cancer cells, ADME studies revealed that it has intrinsically low oral bioavailability because of metabolic enzyme and efflux transporter. This should be further investigated to improve its efficacy in the treatment of apoptotic resistant cancers.^86^

## Quercetin

Quercetin (3, 3′, 4′, 5, 7-pentahydroxyflavone), a natural flavonoid molecule found in fruits, vegetables, leaves, and grains, has anticancer effects linked to its capacity for targeting key molecules, organelles, and tumorigenic pathways (Table 1).^87, 88, 89^ To confirm the involvement of autophagy, Psahoulia *et al.*^66^ treated RAS-transformed colon cells with 3-MA at the early stages, which resulted in the inhibition of vacuolization. Moreover, zVAD-FMK also failed to inhibit vacuolization, showing that the autophagy induced was caspase independent. Treatment of gastric cancer cells induced the vital stages that initiated autophagy progression.^90^ Administration of the inhibitor chloroquine or selective ablation of Atg5 or Beclin-1 using siRNA increased apoptotic cell death, suggesting that autophagy has a protective role against quercetin-induced apoptosis. Functional studies revealed that the activated autophagy is modulated via the Akt-mTOR and HIF-1*α* signaling. Therefore, these xenograft models provided *in vivo* evidence for quercetin-induced apoptosis and autophagy.^90^

Martinez-Outschoorn *et al.*^91^ presented evidence that treatment with quercetin promoted the removal of defective mitochondria from cancer-associated fibroblasts by autophagy /mitophagy that was induced by oxidative stress. As a consequence, the ‘reverse Warburg effect,' whereby these fibroblasts provided nutrients to stimulate mitochondrial biogenesis and oxidative metabolism in adjacent cancer cells, was proposed.

Quercetin treatment induced extensive intracellular vacuolization and phagolysosome formation with accumulation of autophagic biomarkers in epithelial cancer cells, which led to cell cycle arrest and induction of apoptosis.^92^ Before the formation of autophagosomes, inhibition of mTOR activity was observed, accompanied by a marked reduction in the phosphorylation of its substrates, the ribosomal S6 subunit via p70S6 kinase and the eIF4 via its inhibitor 4E-BP1. Inhibition of proteasome activity by quercetin was also observed with accumulation of polyubiquitinated protein aggregates, suggesting that proteasome inhibition is a major cause of the cancer cell death. Therefore, quercetin induces extensive autophagy and subsequent death in cancer cells mediated by the inhibition of proteasomal activity and mTOR signaling.^92^

Li *et al.*^93^ investigated whether autophagy contributes to HSP72-mediated cytoprotection in lipopolysaccharide-induced peritonitis. This is because HSP72 is known to induce autophagy but provide protection against apoptosis. The initial exposure of cultured peritoneal mesothelial cells to lipopolysaccharide resulted first in cell death via autophagy, with subsequent death seen to occur via apoptosis. Therefore, the activation of autophagy acted as a prosurvival mechanism. When autophagy was inhibited by 3-MA or Beclin-1 siRNA, the cells were sensitized to apoptosis, and the antiapoptotic effect of HSP72 was abolished. Also, overexpression of HSP72 enhanced autophagy through JNK phosphorylation and Beclin-1 upregulation. The suppression of JNK activity reversed HSP72-mediated Beclin-1 upregulation and autophagy, which indicated that HSP72-mediated autophagy, is JNK dependent. In the *in vivo* rat model of lipopolysaccharide-induced peritonitis, autophagy was seen to occur prior to apoptosis. When HSP72 was upregulated by geranylacetone, autophagy was increased, whereas apoptosis was inhibited and peritoneal injury reduced. These effects were reversed by downregulation of HSP72 with quercetin. When Li *et al.*^93^ blocked autophagy by chloroquine, there was induction of apoptosis and increased peritoneal dysfunction. Thus, they concluded that HSP72 protects peritoneum from lipopolysaccharide-induced mesothelial cell injury by inducing JNK activation-dependent autophagy and inhibiting apoptosis.

In a study to characterize the bioavailability and metabolic pharmacokinetics of quercetin in rats, 93.8% of the dose was circulating as its sulfates and glucuronides in the bloodstream when administered intravenously and 53% when given orally. These metabolites were seen to be responsible for the *in vivo* effects of quercetin. In both instances, the parent form of quercetin was not detected.^94^

Therefore, treatment with quercetin has numerous anticancer effects, including not only the induction of cell cycle arrest and apoptosis but also of autophagy through modulation of important autophagy signaling pathways such as Akt-mTOR and HIF-1*α* (Figure 3).

## Curcumin

Curcumin (diferuloylmethane), an active ingredient of the spice turmeric *Curcuma longa*, has a potent anticancer effect on cancer cells.^95^ Curcumin targets mainly the PI3K/Akt/mTOR signaling pathway and NF-*κ*B-regulated proteins (Table 1).

Various studies provided evidence that curcumin induced G_2_/M arrest and autophagy in malignant glioma cells through inhibition of the Akt/mTOR/p70S6K and activation of the ERK1/2 pathways, which implied that cell death via autophagy, might be pathway specific.^62, 96, 97^ In a xenograft glioma model, Aoki *et al.*^96^ observed that curcumin induced autophagy and inhibited tumor growth significantly. Shinojima *et al.*^62^ observed that inhibition of NF-*κ*B, which is the main anticancer target of curcumin, does not have a major role in the death of malignant glioma cells. Therefore, autophagy and not NF-*κ*B, is the causal factor of the anticancer effects seen in these cells. Chadalapaka *et al.*^98^ showed that curcumin decreased expression of Sp proteins, whose overexpression in gastric and pancreatic cancers correlates with poor survival and tumor aggressiveness. Downregulation of *EGFR* (a Sp-regulated gene that suppresses autophagy) and decreased phosphorylation of Akt led to the induction of LC3 and cell death in bladder cancer. Curcumin protected HUVECs cells from oxidative stress by inducing autophagy via pI3K/Akt/mTOR and FOXO pathways to interact with Atg7; the involvement of FOXO (a mediator of autophagy) was confirmed in a knockdown study in which autophagy was inhibited by an siRNA.^99^

Curcumin induced autophagy in mesothelioma cells as indicated by increased conversion of LC3-I to LC3-II and formation of autophagosomes, which were reduced by RNA silencing of Atg5.^100^ Mosieniak *et al.*^101^ showed that induction of cellular senescence was accompanied by autophagy in colon cancer cells with an increase in Beclin-1 and p62/SQSTM1 protein levels. Interestingly, the inhibition of autophagy due to diminished expression of Atg5 by RNA interference also decreased the number of cells induced into senescence by curcumin, but did not lead to increased cell death. This study revealed a possible functional link between senescence and autophagy in curcumin-treated cells, a finding that should be investigated further.

Jia *et al.*^102^ presented evidence that curcumin is able to induce both autophagy and apoptosis in a chronic myeloid leukemia cell line via downregulation of the Bcl-2 protein. These effects were confirmed when the potent inhibitor of autophagosome–lysosome fusion, bafilomycin A1 and pan-caspase inhibitor zVAD-FMK suppressed the cell death. In a study on prostate cancer cells, curcumin was shown to induce autophagy cell death through downregulation of another important Bcl-2 family member, Bcl-xL. Curcumin treatment did not induce the cleavage of procaspase-8, -9, -3, or -7 or PARP but led to appearance of the LC3B-II isoform and increased number of autophagosomes.^103^

Treatment of human colon cancer cells with curcumin induced the conversion of LC3-I to LC3-II and degradation of SQSTM1 and autophagosomes.^104^ The autophagic changes induced by curcumin were almost completely blocked in the presence of the antioxidant NAC, indicating that treatment led to ROS production, autophagosome formation, and autolysosomal degradation. The reduction of SQSTM1 degradation by bafilomycin, further confirmed the activation of autophagy cell death.^104^ Kim *et al.*^105^ reported anticancer activity involving ROS in oral squamous cell carcinoma via both apoptosis and autophagy, which was confirmed when NAC blocked autophagic vacuole formation.

Curcumin has been proven to not only induce apoptosis on its own but to also have synergistic effects with various FDA-approved drugs via major inflammatory biomarkers and oncoproteins.^106^ As curcumin promotes autophagy similarly via most of these proteins, therefore this compound is effective in inducing both apoptosis and autophagy in cancer therapy.

However, a major restriction on the use of curcumin as an anticancer agent is its poor absorption, biodistribution, metabolism, and bioavailability. When 400 mg of curcumin were fed to rats, about 60% were found to be absorbed. To address these problems, several formulations have been used, which include nanoparticles, liposomes, micelles, and phospholipid complexes, all with limited success. Also curcumin undergoes metabolism to form various metabolites, such as, glucuronide, sulfate, tetrahydrocurcumin, hexahydrocurcumin, octahydrocurcumin, and hexahydrocurcuminol after oral administration. However, all these metabolites displayed anticancer effects. ^107^

## Resveratrol

Resveratrol (3,5,4-trihydroxystilbene), a natural phytoalexin present in grapes, nuts, and red wine, has chemopreventive properties and multiple mechanisms of action, which may be activated depending on the specific cell type and cellular environment (Table 1).

Opipari *et al.*^63^ provided the first evidence that resveratrol induces cell death through autophagy in five ovarian cancer cell lines, which suggested it as an effective treatment in apoptosis-resistant cells. As autophagy is an adaptive response to nutrient starvation and resveratrol's ability to induce a starvation-like signaling response, that is, reducing the levels of phosphorylated Akt and mTOR to initiate autophagy, is observed in ovarian cancer cells.^108^

Resveratrol was shown to bind to a novel estrogen receptor coactivator, PELP1, and induce its accumulation in autophagosomes.^109^ PELP1 was identified for the first time by a trafficking molecule, hepatocyte growth factor-regulated tyrosine kinase substrate, which binds to it.^110^ The role of hepatocyte growth factor-regulated tyrosine kinase substrate in facilitating the transport of cytoplasmic proteins to autophagosomes for their selective degradation was confirmed in another study involving resveratrol treatment of lung cancer cells.^111^

Puissant *et al.*^29^ presented evidence of resveratrol triggering autophagic death in chronic myeloid leukemia cells via both JNK-mediated p62/SQSTM1 overexpression and AMPK/mTOR activation.^108^ Also resveratrol enhanced the expression of several tubulin subunits, which is important for movement of autophagosomes inside the cell.

Trincheri *et al.*^112^ reported that autophagy can be induced with acute exposure to resveratrol, whereas prolonged exposure activated a caspase-mediated cell death pathway. They observed that genetic inactivation of PI3K, Beclin-1, and Lamp2b inhibited resveratrol toxicity. Beclin-dependent autophagy was confirmed when supplementing the cells with asparagine or knocking down Beclin-1 by RNA interference, abrogated the effect. The effect of Lamp2b was confirmed when its silencing inhibited the fusion of autophagosomes with lysosomes and induced cell viability. Interestingly, zVAD-FMK inhibited cell death but not autophagy. This study uncovered a novel pathway of resveratrol cytotoxicity in which autophagy has two roles, that is, a prosurvival stress response that later in the process, changes to a caspase-dependent apoptotic response. In another study, resveratrol increased ROS level with induction of caspase-8 and caspase-3 cleavage and elevation of LC3-II expression in colon cancer cells; these effects were diminished by NAC.^113^

An interesting non-canonical autophagic process characterized by a Beclin-1/Vps34-independent pathway was observed in MCF-7 cancer cells in response to resveratrol treatment. Overexpression of Bcl-2, which is known to block canonical starvation-induced autophagy by binding to Beclin-1, was unable to reverse the non-canonical autophagy triggered by resveratrol in these breast cancer cells.^8, 114^

SCCA 1, an endogenous cathepsin L inhibitor, is expressed widely in uterine cervical cells. Hsu *et al.*^115^ showed that the cathepsin L–SCCA 1 lysosomal pathway and autophagy were involved in resveratrol-induced cytotoxicity in cervical cancer cells. Inhibition of the autophagic response by wortmannin or asparagine resulted in decreased autophagic death.

Dihydroceramide, an immediate precursor of the apoptotic mediator ceramide in the *de novo* sphingolipid synthesis pathway, was accumulated when resveratrol-induced autophagy occurred as a result of inhibition of dihydroceramide desaturase activity in gastric cancer cells. These effects of resveratrol were mimicked by a dihydroceramide desaturase inhibitor.^116^

Resveratrol-induced autophagy in human glioma cells has the ability to inhibit resveratrol-induced apoptosis.^117^ Autophagy and apoptosis seemed to have different roles, apoptosis causing these cells' death, whereas autophagy delayed apoptosis and protected the cells from death. This suggests that autophagy inhibitors may have the potential to enhance resveratrol antitumor efficacy.^117^

In a large-scale *in vitro* kinase screen, p70 S6 kinase (S6K1) was identified as a target of resveratrol. Blocking S6K1 activity by expression of a dominant-negative mutant or RNA interference was sufficient to disrupt autophagy to an extent similar to resveratrol. Also, co-administration of resveratrol with S6K1 knockdown did not produce an additive effect, which indicated that S6K1 is important for the full induction of autophagy and some of the beneficial effects of resveratrol are due to modulation of S6K1 activity.^118^

SIRT1, one of the best characterized targets of resveratrol, upon activation by this compound induced both autophagy and apoptosis.^119^ However Armour *et al.*^118^ observed that resveratrol decreased autophagy in response to nutrient limitation in multiple cell lines through a pathway independent of SIRT1. Resveratrol is able to induce autophagy in lung cells with cigarette smoke-mediated oxidative stress via regulation of SIRT1 and PARP.^120, 121, 122^ Resveratrol prevented the decline in ATP concentration and SIRT1 expression, as well as the increase in HIF-1*α* expression and autophagy, in the livers of endotoxin-challenged wild-type mice but not in the liver of SIRT1 knockout mice.^123^ These findings provided an insight to the potential roles of SIRT1 and HIF-1*α* expression in systemic inflammation.

Persistent human papillomavirus infection may stabilize ATAD3A (an anti-autophagy factor), inhibit cell autophagy and apoptosis and to increase drug resistance in uterine cervical cancer. Resveratrol's anticancer effects were confirmed by its capacity for reducing ATAD3A expression, increasing abrasion of the mitochondrial outer membrane, and increasing the numbers of autophagosomes.^124^

Resveratrol was shown to trigger autophagic cell death through increased expression of Atg5, 7, 9, and 12 proteins in a human hepatitis C-induced hepatoma cell line.^125^ Also, Filippi-Chiela *et al.*^126^ observed that resveratrol induced the formation of autophagosomes through upregulation of Atg5, Beclin-1, and LC3-II in glioblastoma cells. The PtdIns(3)P effectors, WIPI-1, and WIPI-2 were shown to function downstream during initiation of autophagosome formation. Localization of WIPI-1 at the endoplasmic reticulum and the plasma membranes upon the induction of autophagy confirmed its involvement with the autophagosomal membrane.^127^ In another study, WIPI-1 specifically bound PtdIns(3)P, accumulated at the phagophore, and become a membrane protein of the autophagosome generated. WIPI-1 was observed to function upstream of both Atg7 and 5 and stimulated an increase of LC3-II upon nutrient starvation. These findings constituted evidence that resveratrol-mediated autophagy was via the non-canonical pathway, Beclin-1 independent but Atg7 and 5 dependent.^128^

As seen with curcumin, resveratrol can induce premature senescence that is associated with a blockade of autolysosome formation, as assessed by the absence of colocalization of important markers of autophagosomes and lysosomes, LC3 and Lamp-2, respectively. Resveratrol was also able to downregulate the level of Rictor, a vital component of the mTORC2 complex, which led to decreases in RhoA-GTPase activity, altered actin cytoskeleton network and increases in senescence-associated *β*-gal activity. Therefore, resveratrol has the ability to attenuate the autophagic process via downregulation of Rictor, which may be the mechanism of tumor suppression associated with premature senescence.^129^

Resveratrol upon administration is readily absorbed and metabolized as glucuronides and sulfates and the oral bioavailability of resveratrol is almost zero owing to its rapid metabolism activity.^130^ However, as resveratrol has shown versatility in inducing autophagy in numerous cancer types and targeting a wide array of autophagy-associated proteins, further clinical investigations are important to fully evaluate the efficacy and bioactivity of resveratrol in the killing of cancer cells via autophagy.

## Conclusion

One of the most important unsolved problems in cancer therapy is increased tumor resistance to treatment, be it chemotherapy, radiotherapy, or any of the targeted therapies. This increased resistance is a direct effect of defects in apoptosis. An alternative form of cell death, namely, autophagy, may be the ultimate solution for this problem. The evidence presented here (Table 1 and Figure 2) demonstrates that several phytochemical polyphenolic compounds found in foods are able to mediate both canonical and non-canonical autophagy via multiple pathways targeting important proteins in a number of cancer types. Understanding their mechanisms is of vital importance in ensuring cell death *versus* survival. These polyphenolic compounds have the ability to induce both apoptosis and autophagy, thereby maximizing death of cancer cells. As they are food compounds, furthermore, they may offer greater safety, both through their inherently lower toxicity and through allowing reduction of doses and side effects as compared with synthetic drugs. Combining FDA-approved drugs with known polyphenolic compounds such as rottlerin, genistein, quercetin, curcumin, and resveratrol may provide novel therapeutic strategies in the treatment of cancer to combat the substantial problem of drug resistance in cancer therapy.

## Figures and Tables

**Table 1:** Effect of polyphenols on induction of autophagy in cancer

**Treatment**	**Cancer cell/tissue**	**Mechanism**	**Dose**		**Reference**
			***In vitro* (μM)**	***In vivo* (mg/kg)**	
Rottlerin	Pancreatic	PKCδ/TG2	2–4	—	^12, 13^
	Fibrosarcoma	PKCδ-indep	0.5–10	—	^131^
	Breast	mTORC1	3	—	^77^
Genistein	Rat hepatocytes	Cytokeratin	—	100	^64, 65^
	Ovarian	Akt	25–100	—	^82^
	Ovarian	PDE4A4& p62/SQSTM1	10	—	^83^
	Lung	N-CoR/Hsc70	25–50	—	^85^
Quercetin	Colon	Ras	20	—	^66^
	Fibroblast-breast	ROS	10	—	^91^
	Gastric	Akt/mTOR&HIF-1α	10–160	50	^90^
	Rat mesothelial	HSP72/JNK&Beclin-1	—	100	^93^
	Breast, Cervical, Ovarian	mTOR/eIF4E-BP1/p70S6K	30–90	—	^92^
Curcumin	Brain	Akt/mTOR/p70S6K	10–50	100	^62, 96^
		ERK1/2			
	CML	Bcl-2	5–20	—	^102^
	Bladder	Akt	40	—	^98^
	Prostate	Bcl-xL	10–50	—	^103^
	Colon	ROS	10–40	—	^104^
	Brain	PI3K/Akt/mTOR	2	300	^97^
	Mesothelioma	ND	10–50	—	^100^
	Oral	ROS	10	—	^105^
	Colon	Beclin-1&p62/SQSTM1	10	—	^01^
	Endothelial	PI3K/Akt/mTOR&FOXO1	1–10	—	^99^
Resveratrol	Ovarian	ND	50	—	^3^
	Salivary gland	PELP1/HRS	50–100	—	^110^
	Ovarian	Akt/mTOR/ p70S6K	25–100	—	^108^
	Lung	PELP1/HRS	50–100	—	^111^
	Colorectal	PI3K/Beclin-1/Lamp2b	100	—	^112^
	Breast	Akt/PKB/mTOR/p70S6K	64	—	^132^
	Cervical	Cathepsin L	100	—	^115^
	Gastric	Dihydroceramide desaturase	50	—	^116^
	Brain	Beclin-1	150	—	^117^
	Fibroblast, Cervical	p70S6K	50–200	—	^118^
	CML	JNK/p62, AMPK/mTOR	10–50	—	^133^
	Lung	SIRT1/PARP-1	10	—	^120^
	Hepatoma	ND	20	—	^125^
	Liver	SIRT1, AMPK, HIF-1*α*	—	20	^123^
	Colon	SIRT1	100	—	^122^
	Brain	ND	30	—	^126^
	Cervical	ATAD3A	30–100	—	^124^
	Osteosarcoma, melanoma, cervical, breast	WIPI-1	64	—	^128^
	Colon	ROS	25–150	—	^113^
	Skin	mTORC2 (Rictor)	50	—	^129^

Abbreviations: Akt/PKB, protein kinase B; AMPK, adenosine monophosphate-activated protein kinase; ATAD3A, ATPase family AAA domain containing 3A; Bcl-2, B-cell lymphoma 2; eIF4E-BP1, eukaryotic initiation factor 4E binding protein 1; ERK 1 / 2, extracellular signal-regulated kinases 1 / 2; HIF-1α, hypoxia-inducible factors -1 alpha; HRS, hepatocyte growth factor-regulated tyrosine kinase substrate; HSP72, heat shock protein 72; JNK, c-Jun N-terminal kinases; Lamp 2b, lysosome associated membrane proteins 2b; mTOR, mammalian target of rapamycin; mTORC 1 / 2, mTOR complex 1 / 2; N-CoR, nuclear receptor corepressor; ND, not determined; p62/SQSTM1, the ubiquitin-binding protein p62 or sequestosome 1; p70S6K, p70S6 kinase; PARP-1, Poly [ADP-ribose] polymerase 1; PDE4A4, cyclic AMP phosphodiesterase-4A4; PELP1, proline-, glutamic acid-, and leucine-rich protein-1; PI3K, phosphatidylinositol 3-kinases; PKCδ, protein kinase C delta; Ras, rat sarcoma protein; ROS, reactive oxygen species; SIRT1, Sirtuin 1; Tcf-4, transcription factor -4; TG2, tissue transglutaminase; TSC1/2, tuberous sclerosis 1 / 2; Vps 34/ Class III PI 3-kinase; WIPI, WD-repeat protein interacting with phosphoinosides; Wnt, wingless integration signaling pathway. Induction of autophagic death by the five dietary polyphenolic compounds rottlerin, genistein, quercetin, curcumin and resveratrol in various cancer types, the regulatory mechanisms involved and the effective doses for *in vitro* as well as *in vivo* analyses

## Abbreviations

3-MA: 3-methyl adenine
4E-BP1: eIF4E binding protein 1
ADME: absorption, distribution, metabolism and excretion
AMPK: AMP-dependent protein kinase
ATAD3A: ATPase family AAA domain containing 3A
ATG: autophagy regulated genes
ATM: ataxia telangiectasia mutated
CML: chronic myeloid leukemia
eIF4: eukaryotic initiation factor of protein biosynthesis
ER: endoplasmic reticulum
ERK: extracellular signal-regulated kinase
FDA: Food and Drug Administration
FOXO: forkhead box class O
HIF-1*α*: hypoxia-induced factor 1*α*
HRS: hepatocyte growth factor–regulated tyrosine kinase substrate
HSP72: heat shock protein 72
HUVECs: human umbilical vein endothelial cells
JNK c-Jun: *N*-terminal kinase
Lamp2b: lysosome-associated membrane proteins 2b
MAPK: mitogen-activated protein kinase
MDC: monodansylcadaverin
mTOR: mammalian target of rapamycin
NAC: N-acetylcysteine
N-CoR: nuclear receptor co-repressor
NF-κB: nuclear factor kappa B
NSCLC: non-small cell lung carcinoma
PARP: poly(ADP ribose) polymerase
PCD: programmed cell death
PELP1: proline-, glutamic acid–, and leucine-rich protein-1
PDE4A4: cyclic AMP phosphodiesterase-4A4
PI3K/Akt: phosphatidylinositide 3-kinase
PKB: protein kinase B
PKCδ: protein kinase C delta
ROS: reactive oxygen species
SCCA: squamous cell carcinoma antigen
siRNA: small interfering RNA
SIRT1: sirtuin 1
SQSTM1: p62/sequestome 1
TG2: transglutaminase 2
TSC2: tuberous sclerosis complex 2
WIPI-1/2: Wd-repeat proteins interacting with phosphoinositides
zVAD-fmk: benzyloxycarbonyl-Val–Ala–Asp fluoromethylketone
